# The interpersonal theory of suicide in the understanding of eating disorders

**DOI:** 10.1192/j.eurpsy.2025.1398

**Published:** 2025-08-26

**Authors:** C. Gramaglia, R. Calati, B. Aldrovandi, F. Turolla, V. Zanoli, M. Probo, P. Longo, A. Caldiroli, G. Abbate Daga, M. Clerici, P. Zeppegno

**Affiliations:** 1 Medicina Traslazionale, Università del Piemonte Orientale; 2 Maggiore della Carità University Hospital, Novara; 3 University of Milan-Bicocca, Milan, Milano, Italy; 4 Department of Adult Psychiatry, Nîmes University Hospital, Nimes, France; 5 Università del Piemonte Orientale; 6 Dipartimento Salute Mentale SUD – ASL Novara, Novara; 7 Neuroscience, University of Turin, Centro Esperto Regionale per i Disturbi del Comportamento Alimentare, Torino; 8 Mental Health, Fondazione IRCCS San Gerardo dei Tintori, Monza, Italy

## Abstract

**Introduction:**

Suicidal behavior could be one of the declinations of destructiveness in patients with Eating Disorders (EDs), and represents an important cause of death in these patients. In recent years some theoretical models aimed at a deeper undestanding of suicidal behaviors have been developed and proposed, including the Interpersonal Theory of Suicide (IPTS) by Thomas Joiner, and it has been applied to EDs patients with mixed results.

**Objectives:**

The objective of this study was to deepen the understanding of the problem of suicide in EDs, with the aid of the IPTS.

**Methods:**

Multicenter study coordinated by the Department of Psychology - University of Milano-Bicocca (UniMiB) and the Psychiatry of the Università del Piemonte Orientale (UPO); the recruitment of patients with EDs involves: Novara Mental Health Center and Psychiatry outpatient clinic of the Maggiore della Carità University Hospital in Novara, Regional Expert Center for the Diagnosis and Treatment of Eating Disorders in Turin, outpatient clinic for Nutrition and Eating Disorders, IRCCS San Gerardo dei Tintori Foundation in Monza. Healthy controls were enrolled through UPO and UniMiB. Variables related to suicidal ideation and suicide attempts, IPTS constructs and psychological factors (anxiety, somatic and mental pain, reasons for living, enteroceptive awareness, self-esteem, body image, etc.) were assessed.

**Results:**

Preliminary analyses involving 28 EDs patients (ED group) and 45 healthy controls (HCs). The ED group reported increased lifetime and past-two-weeks suicidal ideation and a greater number of lifetime suicide attempts. With regard to IPTS, a higher Perceived Burdensomeness (INQ-PB) emerged in the ED group compared to HCs; no differences emerged in Thwarted Belongingness and in the Acquired Capability for suicide. In the EDs group, state and trait anxiety, mental pain, Visual Analogue Scale scores for somatic and psychological pain are higher; self-esteem is lower. Correlations emerged between INQ-PB, mental pain and self-esteem in EDs patients: as mental pain increases and self-esteem decreases, INQ-PB increases. The EDs sample has currently almost doubled, and new analyses will be performed to see whether the findings described above are confirmed in a larger sample; analyses comparing different subtypes of EDs will be run as well.

**Conclusions:**

With the limitations of the sample size recruited in the pilot phase, which we aim to overcome by running new analyses in a larger sample as recruitment is ongoing, the preliminary data of this study offer preliminary support to the existing data in the literature on IPTS in EDs. An increase in IPTS construct scores appears to be independent of the severity of body image disorder in these patients.

**Disclosure of Interest:**

None Declared

